# Role of G Protein-Coupled Receptors in Control of Dendritic Cell Migration

**DOI:** 10.1155/2014/738253

**Published:** 2014-03-10

**Authors:** Yuan Liu, Guixiu Shi

**Affiliations:** Department of Rheumatology and Clinical Immunology, The First Affiliated Hospital, Xiamen University, No. 55, Zhenhai Road, Xiamen 361003, China

## Abstract

Dendritic cells (DCs) are highly efficient antigen-presenting cells. The migratory properties of DCs give them the capacity to be a sentinel of the body and the vital role in the induction and regulation of adaptive immune responses. Therefore, it is important to understand the mechanisms in control of migration of DCs to lymphoid and nonlymphoid tissues. This may provide us novel insight into the clinical treatment of diseases such as autoimmune disease, infectious disease, and tumor. The chemotactic G protein-coupled receptors (GPCR) play a vital role in control of DCs migration. Here, we reviewed the recent advances regarding the role of GPCR in control of migration of subsets of DCs, with a focus on the chemokine receptors. Understanding subsets of DCs migration could provide a rational basis for the design of novel therapies in various clinical conditions.

## 1. Introduction

Migration of immune cells is a fundamental biological process involved in normal physiology. This process increases the chance that lymphocytes will encounter the antigen and is also critical to the development of an inflammatory response. Abnormal immune cells migration is always associated with the development and progression of autoimmune diseases [1–3]. Many studies have provided strong support for this idea, and clinical studies have indicated that pharmacological inhibitors on immune cells migration can be highly effective in certain disease conditions [4, 5].

Dendritic cells (DCs) are highly efficient antigen-presenting cells (APC). Several subsets of DCs exist in mice and humans with distinct immunological activities, tissue distribution, and migratory properties. Following uptake of Antigen and in response to inflammatory signals, DCs reside within peripheral tissue become mature and migrate to lymph nodes where they initiate the acquired immunity [6]. In addition to activating the immune response, DCs are also decisive in creating tolerance [7]. The migratory properties of DCs give them capacity to be a sentinel of the body in recognizing the alloantigens, xenoantigens, autoantigens, and neoantigens, and give them the vital role in the induction and regulation of adaptive immune responses. Therefore, it is important to understand the mechanisms in control of migration of DCs, which may provide us novel insight into the clinical treatment of diseases such as autoimmune disease, infectious disease, and tumor.

G protein-coupled receptors (GPCR), the 7 trans-membrane receptors, encoded by more than 800 genes, are activated by a large variety of factors ranging from small amines to hormones and chemokines [8]. Chemokines are the most well-known factors in the induction of immune cell migration, and all chemokine receptors identified so far are membrane-bound GPCRs. Besides chemokines, several bioactive lipids or hormones such as Sphingosine 1-phosphate (S1P), Lysophosphatidic acid (LPA), and angiotensin II can also regulate the migration of DCs by binding to receptors coupled to G proteins [9–11]. GPCRs play a central role in control of DCs migration. In this review, we focused on the role of chemokine receptors in control of migration of DCs.

## 2. Subsets of Dendritic Cells and Routes of Dendritic Cells Trafficking

Definition of subsets of DCs is still evolving with new technology. Dendritic cell was first discovered in peripheral lymphoid organs in the late 1970s [12]. Soon after that, DCs populate in nonlymphoid tissue such as Langerhans cell (LC) were identified. Classical mouse DCs in lymphoid tissue were further subdivided into two subsets by the presence or absence of CD8 expression (CD8^+^ and CD11b^+^ cDCs), and DCs in nonlymphoid tissue were subdivided by the expression of integrin CD103 (CD103^+^ CD11b^−^ and CD11b^+^ cDCs) [13, 14]. However, human DCs did not express CD8, they can be split into CD1c^+^ and CD141^+^ subsets which share homology with mouse classical DCs expressing either CD11b (CD1c^+^ DCs) or CD8/CD103 (CD141^+^ DCs) [15].

In recent years, a population of DCs which morphologically resemble plasma cells but, upon exposure to viral stimuli, produce enormous amounts of interferon (IFN)-*α*, was identified and named as plasmacytoid dendritic cells (pDCs). The definition of classical DCs (cDCs) then came out which referred to DCs other than pDCs. DCs can also be classified into myeloid DCs and lymphoid DCs based on their origin. Phenotype characteristics and immune function of subsets of DCs were summarized by Merad et al. [16]. Subsets of DCs are different in migratory routes.

**Figure 1:**
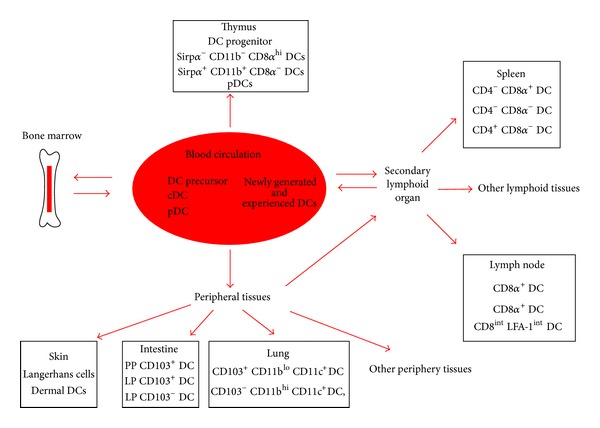
Schematic illustration of routes of migration of mouse DCs. DC precursors are released from the BM into the blood; DC progenitors can also be found in multiple locations including the thymus, blood, lymph, and most visceral organs. DC precursors seeded peripheral lymphoid tissues and nonlymphoid tissues and differentiated them into committed DCs. cDCs in peripheral tissues can access afferent lymph upon receiving a mobilization signal and travel to the draining LNs during both inflammation and steady-stated. pDCs travel to the LNs and spleen via hematogenous route. Some DCs might exit lymph nodes (LN) and start a still undefined pathway to recirculate. The circulating DCs in the blood contain both DC precursors and differentiated DC subsets, which are a mixture of newly generated cells from the BM and experienced DCs which have reentered the circulation from peripheral tissues.

Most of the conclusions about the migration of DCs came from researches on mouse DCs. All DCs are thought to be ultimately derived from bone marrow (BM) [17]. Many DCs begin their journey with their release from the BM into the blood. Circulating DCs in the blood contain both DC precursors and differentiated DC subsets, which are a mixture of newly generated cells from the BM and experienced DCs. DCs in the blood subsequently traffic to lymphoid and nonlymphoid tissues. DCs in peripheral tissues such as skin, lung, and intestine migrate to draining lymph nodes to initiate acquired immunity during inflammation state, and this migration also happens in steady-state. A small fraction of these experienced DCs can reenter into the blood circulation and begin another cycle of journey. Unlike cDCs, pDCs are rarely found in peripheral tissues except for the intestine and enter the LN via the high endothelial venules (HEV) instead of afferent lymphatics. pDCs have been reported to migrate to sites of inflammation and to infiltrate tumors as well as solid organ transplants [18]. The routes of migration of mouse DCs are schematic illustrated in Figure 1.

## 3. Chemokine Receptors and DCs Migration

Chemokines are small molecular weight chemoattractant peptides first identified and characterized as being induced at sites of inflammation and thought to orchestrate the influx of leucocytes to the inflamed tissue. To date, more than 40 chemokines have been identified and classified into four families, C, CC, CXC, and CX3C, according to the motif displayed by the first two of four cysteine residues at the N terminus of the molecule [19]. The specific effect of chemokines is mediated by their G protein-coupled receptors. DCs migration is largely mediated by the interaction of chemokines with their G protein-coupled receptors. Selective expression of chemokine receptors on DCs tightly regulates the normal and inflammatory trafficking within lymphoid and nonlymphoid tissues [20].

There are 18 chemokine receptors that have been identified so far, receptors for fractalkine (CX3CR1) and lymphotactin (XCR1), 5 receptors that selectively bind certain CXC chemokines named as CXCR1 to CXCR5, 9 receptors which bind to CC chemokine named as CCR1 to CCR9, D6 which has been termed as CCR10 by some research group, and receptor which can bind to both CXC and CC chemokines known as the Duffy antigen receptor for chemokines (DARC) [21].

Studies on the in vitro derived DCs (CD34^+^ stem cell or bone marrow-derived DCs) found that immature DCs can express CCR1, CCR2, CCR5, CCR6, CXCR1, CXCR2, and CXCR4, with these expression patterns differing somewhat among DC subsets. However, chemokine receptors expression profile from the in vitro study may not accurately mirror the changes that occur on DCs in vivo. It has been indicated that several chemokine receptors including CCR1, CCR2, CCR3, CCR5, CCR6, CCR7, CCR9, CCR10, CXCR3, CXCR4, CXCR5, and ChemR23 are involved in control of different subsets of DCs recruitment to periphery tissues and migration to secondary lymphoid tissues or migration within lymphoid tissues.

### 3.1. Chemokine Receptors and Mouse DCs Migration

#### 3.1.1. CCR1

Expression of CCR1 can be detected on immature DCs. Its ligand CCL9, also known as MIP-*γ*, is constitutively secreted by the follicle-associated epithelium (FAE) within the dome regions of the Peyer's patches in mouse, and it may play a role in the recruitment of CD11b^+^ DCs in the dome regions of the Peyer's patches in addition to the CCR6-dependent manner [22]. Study also indicated the possible role of CCR1 as well as CCR5 in regulation of recruitment of immature DC precursors into resting airway tissues and during acute bacterial-induced inflammation by using Met-RANTES, which retained high binding affinity to CCR1 and CCR5. However, the effect of CCR1 in control of DCs migration appeared to depend on the nature of the eliciting stimulus, because the recruitment of DCs was not affected by Met-RANTES in inflammation induced by Sendai virus infection and after aerosol challenge of sensitized animals with the soluble recall Ag OVA [23]. These data suggest that CCR1 might play a role in recruitment of immature DCs to periphery tissues during both steady-state and inflammatory-state. However, all of these conclusions came from some indirect data, the direct effect of CCR1 in the DCs migration still needs to be proved.

#### 3.1.2. CCR2

CCR2 is expressed on immature DCs, and its expression can be also detected in mature DCs [54]. Its main ligand is CCL2 [55]. Several studies demonstrated the central role of CCR2 in the recruitment of DCs to the lung during inflammation by using CCR2 knockout mice. Robays and his colleague showed that CCR2 was involved in the recruitment of DCs in the lung during allergic inflammation and may mediate the release of DC precursors into the bloodstream, and CCR2 was critical in inducing Th2 responses [24, 25]. However, controversy existed about the role of CCR2 in the Th1 or Th2 induction. In the situation of fungal pathogen and mycobacterium tuberculosis infection, CCR2 was shown to be involved in recruitment of myeloid DCs and CD11c^dim/intermediate^ DCs to the lung, respectively, and it was supposed to mount Th1 immune responses [26, 27]. Besides the role of CCR2 in the immature DCs trafficking, it can also regulate the migration of some activated DCs to the draining LNs. Study using CCR2-null mice showed that migration of Langerhans cell from skin to draining lymph nodes was impaired with reduced Th1-inducing DCs (CD8*α* ^+^ DC) in the spleen and impaired infection-induced relocalization of CD11c^+^ DC from the marginal zone (MZ) to the T cell areas in spleen [28].

#### 3.1.3. CCR5

CCR5 is the major HIV-1 coreceptors for R5 strains. CCR5 is shown to be expressed by immature blood DCs in human, and in vitro maturation of blood DCs resulted in median 3-fold increases in CCR5 expression [56]. Its ligands CCL3, CCL4, and CCL5 are produced in the inflamed LNs of humans and/or mice [57, 58]. In mice, the role of CCR5 in the migration of pDCs to LNs has been demonstrated by several studies. By using CCR5 deficient pDCs (derived from BM of CCR5 −/− mice), Diacovo proved that migration of pDCs from blood to inflamed peripheral lymph nodes relied in part on CCR5 rather than CXCR3 [29].

#### 3.1.4. CCR6

CCR6 is expressed by immature DCs, different in subsets of DCs (absent from CD8*α* ^+^ lymphoid DC), and the expression level of CCR6 decreases progressively as DCs mature [59]. Its ligand CCL20 is expressed by inflamed skin, mucosal epithelium, and mucosal-associated lymphoid tissue epithelium, and it plays an important role in recruitment of immature DCs to inflamed skin or mucosa [20]. Study on the CCR6 deficient mice showed that myeloid CD11c^+^ CD11b^+^ dendritic cells were absent from the subepithelial dome of Peyer's patches, which indicated the role of CCR6 in recruitment of myeloid DCs to the Peyer's patches [30, 31]. Similarly, studies also demonstrated the role of CCR6 in recruitment of myeloid DCs to the inflamed epithelial tissues such as skin [32]. Study also indicated that in some situation such as consecutive to an initial CCR7-mediated recruitment from blood into lymphoid tissues draining inflamed epithelia, pDCs might be conditioned to acquire CCR6 and CCR10 expression and migrate into inflamed epithelia of mucosae or skin. This study suggests an unexpected pDCs migratory model, after CCR7-mediated extravasation of blood pDCs into lymphoid tissues draining inflamed epithelia, they may be instructed to up-regulate CCR6 and/or CCR10 allowing their homing into inflamed epithelia (in mucosae or skin) [33].

#### 3.1.5. CCR7

CCR7 has been identified as a key regulator of lymphocytes trafficking to secondary lymphoid organs [60]. It is expressed by mature DCs [61]. CCL19 and CCL21 (also known as secondary lymphoid-tissue chemokine) are the two ligands of CCR7 which are found to express in the afferent lymphatic vessel and LN paracortex and subcapsular sinus (SCS) in mice [62]. The role of CCR7 in DCs migration has been well studied in mice DCs. Several studies demonstrated the role of CCR7 in control of tissue-resident myeloid dendritic cells from periphery tissues such as skin and mucosa migration to draining LNs via the afferent lymphatics under inflammatory and steady-state conditions [34, 63]. The role of CCR7 in migration of pDCs remains controversial. Some studies showed that murine pDCs were CCR7 negative or low, and functionally were considered unresponsive to CCR7 ligands [64, 65]. However, studies using CCR7-deficient mice demonstrated the role of CCR7 in regulation of pDCs migration to secondary lymphoid organs. Sebastian found that ex vivo derived nonstimulated and naive pDC express CCR7, CCR7-deficient pDC showed impaired homing to resting as well as inflamed LN, and identified that CCR7 was an important LN homing receptor for pDC under both steady-state and inflammatory conditions [35]. Umemoto showed that CCR7 as well as CXCR4 were both critical chemokine receptors required for pDCs to migrate into white pulp in the spleen under steady-state conditions [36].

#### 3.1.6. CCR9

CCR9 has first been identified on T cells as a chemokine receptor that directs these cells to migrate to the intestine. The CCR9 receptor is not unique to T cells and has also been reported on both myeloid and pDCs, and the expression level of CCR9 was inversely related to the maturation state of DCs [66]. CCL25, also known as thymus-expressed chemokine (TECK), is the ligand of CCR9, which is found in the thymic cortex and in the small intestinal mucosa [67, 68]. It was reported that CCR9 controlled the migration of pDC to the small intestine under both steady-state and inflammatory conditions [37]. The CCR9^+^ pDCs in tissue was thought to be immunosuppressive population [69, 70]. However, the role of CCR9 in myeloid DCs migration still needed to be investigated.

#### 3.1.7. CXCR3

Study using the human CXCR3-specific monoclonal antibodies showed that CXCR3 was expressed in certain dendritic cells subsets, specifically myeloid-derived CD11c^+^ cells both in normal lymphoid organs and inflammatory conditions [71]. Study also showed that CXCR3 was functionally expressed in pDCs and induced migration of pDCs. By using CXCR3 (−/−) mice, Yoneyama et al. demonstrated that CXCR3 played an important role in recruitment of pDC precursors to inflamed lymph nodes through high endothelial venules (HEV) in propionibacterium acnes-primed or HSV-infected mice [38]. And similarly, Asselin-Paturel showed that murine CMV infection and systemic injection of TLR7 and TLR9 ligands can induce migration and clustering of splenic pDCs in the spleen marginal zone, which was dependent on CXCR3 [39]. However, in the study of Diacovo, it was showed that CCR5 instead of CXCR3 was required for pDC migration in response to heat-killed mycobacterium tuberculosis. This difference might be due to the different inflammatory conditions [29].

#### 3.1.8. CXCR4

CXCR4 is the major HIV-1 coreceptors for X4 HIV-1 strains. The expression of CXCR4 on immature DCs is low and is up-regulated during maturation [56]. Its ligand CXCL12 is one of the three most important chemokines (CCL19, CCL21, and CXCL12) which directs DCs migrate from sites of infection to secondary lymphoid organs. Kabashima found that CXCR4 was highly expressed on migrated cutaneous DCs and its ligand, CXCL12, was detected in the LYVE-1(+) lymphatic vessels in the skin. By using CXCR4 antagonist 4-F-Benzoyl-TN14003, they demonstrated that CXCR4 was required for migration of cutaneous dendritic cells to LNs [40]. Umemoto showed that CXCR4 as well as CCR7, cooperatively regulated pDCs migration to the splenic white pulp under steady-state conditions [36].

#### 3.1.9. CXCR5

It was thought that expression of CXCR5 was restricted to mature, recirculating B cells as well as small subpopulations of CD4^+^ and CD8^+^ T lymphocytes [72]. Study also indicated that CXCR5 can be expressed by activated DCs and may be involved in their migration to draining LNs. The CXCR5 ligand CXCL13, also known as B lymphocyte chemoattractant or (BLC), is highly expressed in B cell zones of secondary lymphoid organs. Saeki showed that activated dermal type DCs expressed CXCR5 and these DCs utilize CXCR5 and BLC as a possible mechanism to migrate to B cell zones as well as T cell zones (TCZ) in draining LN in vivo. However, in vitro murine bone marrow derived DCs displayed less CXCR5 expression than the activated skin DCs, and they do not migrate to BLC [41, 73].

#### 3.1.10. ChemR23

ChemR23 is a previously orphan protein G coupled receptor highly expressed in immature pDCs and at lower levels in myeloid DCs. Chemerin is the natural ligand of the ChemR23 and a chemoattractant factor for human immature dendritic cells (DCs), macrophages, and NK cells [74]. It played a central role in human pDCs migration. It was reported that ChemR23 was not present on mouse DCs [75]. However, Souphalone demonstrated that ChemR23 was functionally expressed by mouse DCs and mediated an anti-inflammatory activity in a lung disease model [74]. These controversies on the expression of ChemR23 on mouse DCs are presumably the result of the different sensitivity of the Abs used in these studies.

### 3.2. Chemokine Receptors in Human DCs Migration and Their Role in Diseases

Studies on the role of CCRs in human DCs migration are relatively few compared to studies in mouse. Most conclusions came from indirect evidence by using specific antibody or by analyzing their expression to speculate their role in human DCs migration.

Sato et al. indicated the role of CCR1 and CCR3 in human peripheral blood monocyte-derived dendritic cells migration by using monoclonal antibody (MoAb) to CCR1 and CCR3 [42]. Human cytomegalovirus may use a mechanism by down-regulating CCR1 and CCR5 expression on human DCs to paralyze the early immune response of the host [76], and filarial infection can also down-regulate the CCR1 expression on monocyte-derived DCs which may alter DCs migration [77].

By analyzing the expression of CCR2 and CCR6 on subsets of DCs as well as the ligands of CCR2 and CCR6 expression in different sites in the body, Vanbervliet indicated the possible role of CCR2 and CCR6 in control DCs migration by raising a novel model of how DCs in the blood migrate to inflamed epithelial surfaces: CCR2(+) circulating DC or DC precursors are mobilized into the tissue via the expression of MCP by cells lining blood vessels, and these cells traffic from the tissue to the site of pathogen invasion via the production of MIP-3alpha/CL20 by epithelial cells and the up-regulation of CCR6 in response to the tissue environment [43].

CCR3 is the chemokine receptor initially discovered on eosinophils. Study showed that it was also expressed by human DCs that differentiated from blood monocytes, DCs that emigrated from skin (epidermal and dermal DCs), and DCs derived from CD34^+^ hemopoietic precursors in bone marrow and umbilical cord blood. Unlike other chemokine receptors, such as CCR5 and CCR7, the expression of CCR3 is not dependent on the state of maturation. Indirect study by using CCR3 antibodies indicated the possible role of CCR3 in the DCs migration induced by its ligand eotaxin and eotaxin-2 [42, 44]. Studies on the role of CCR3 in the DCs migration are few, and the specific role of CCR3 in control of subsets of DCs migration is still not clear.

In human, the role of CCR5 has also been indicated in DCs migration in some situations such as in HIV-1 infection and Acute Graft-Versus-Host Disease [45–47]. However, unlike mice pDCs, the recruitment of pDCs appeared to be CCR5 independent. Pashenkov showed that expression of CCR5 was elevated on blood myeloid (CD11c^+^) DC in multiple sclerosis (MS) and optic neuritis patients compared to noninflammatory controls, its ligands RANTES and MIP-1beta were expressed in MS lesions, and the expression of CCR5 by myeloid DC in blood correlated with numbers of these cells in cerebrospinal fluid (CSF), which suggest that CCR5 may contribute to recruitment of myeloid DC (CD11c^+^) to the CSF in these patients, but recruitment of plasmacytoid DC to CSF appeared to be CCR5-independent [46].

Studies also showed that CCR6 was expressed on leukemic pDCs and blood pDCs from melanoma patients and involved in the recruitment of pDC to lesions of skin [48, 49].

Most of the conclusions about the role of CCR7 on the human DCs migration came from the research on mouse DCs. The specific role of CCR7 in subsets of human DCs migration still needed to be confirmed as in mouse DCs.

It was found that in some inflamed situations such as in psoriatic lesions, pDCs found in the lesions were nearly all CXCR3(+), indirectly implicated the possible role for CXCR3 in mediating the recruitment of pDCs into the periphery tissue and developing lesions in human [50]. Besides its ligand CCL9–11, research about uveitis indicated that CXCR3 was involved in the immature DCs migration induced by retinal autoantigens S-antigen (S-Ag) and interphotoreceptor retinoid binding protein (IRBP), suggesting its role in the autoimmune disease [51].

In human DCs, it was found that ChemR23 was expressed both on pDCs and myeloid DCs. Its ligand can induce the transmigration of plasmacytoid and myeloid DCs across an endothelial cell monolayer in vitro. The Chemerin (+) endothelial cells were found to be surrounded by ChemR23(+) pDCs, which suggest a key role of the ChemR23/Chemerin axis in directing plasmacytoid DC trafficking [52]. Similarly, De Palma found that Chemerin was associated with tubular epithelial cells and renal lymphatic endothelial cells in patients with lupus nephritis but not in normal kidneys, and ChemR23-positive DCs had infiltrated the kidney tubulointerstitium in patients with severe lupus nephritis. The induced Chemerin can result in an efficient transendothelial migration of pDCs measured in transwell systems. These data suggest the role of ChemR23 in the recruitment of pDCs within the kidney in lupus nephritis patients [53].

**Figure 2:**
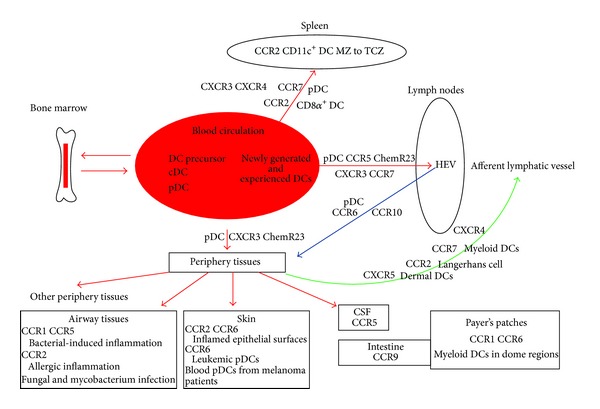
Chemokine receptors involved in migration of mouse and human DCs subsets. CCR7, CXCR4, CCR2, and CXCR5 are involved in subsets of cDCs migration from periphery tissues to draining LNs in both inflammation and steady-state. CCR5, ChemR23, CXCR3, and CCR7 are involved in migration of pDCs to LNs via hematogenous route. CCR2 is implicated in control of CD8*α* ^+^ DC to the spleen and relocalization of CD11c^+^ DC from the marginal zone to the T cell areas in spleen. CCR7, CXCR3, and CXCR4 are shown to be involved in pDCs migration from blood to spleen. CCR1, CCR2, CCR5, and CCR6 are involved in the recruitment of cDCs to different tissues in specific situations. CCR9 is shown to have a role in controlling the migration of pDC to the small intestine under both steady-state and inflammatory conditions. In other situations of inflammation or tumor, CXCR3, ChemR23, and CCR6 are implicated to be involved in pDCs migration to periphery tissues.

The role of chemokines receptor in control of subsets of mouse and human DCs migration was summarized in Tables 1 and 2 and Figure 2.

## 4. Signaling Pathways Involved in Chemokine Receptor Signaling

All chemokine receptors are thought to couple to G proteins. The heterotrimeric G-proteins consist of a *α*-subunit that binds and hydrolyses GTP as well as a *β*- and a *γ*-subunit that form an undissociable complex. Based on the types of their *α* subunits, G proteins can be grouped into four subfamilies, they are G*α*i, G*α*s, G*α*q/11, and G12/13, each subfamily contains several members of G proteins [78].

The mechanism involved in the CCR7 signaling has been well studied; it is a multimodule model with the involvement of G*α*i, G*α*q, and G*α*12 [79]. It was thought that chemotaxis induced by chemokine receptors was mainly through the G*α*i subfamily. The ligation of CCR7 and its ligands mediated the activation of G proteins induced by the binding of GTP to G*α*i and the release of free *βγ* subunits. The *βγ* subunits subsequently activated downstream effectors such as PI3K which regulate the Akt pathway [80]. However, it seemed that these enzymes did not regulate either chemotaxis or the speed of DCs but regulated CCR7-dependent DC survival [81, 82]. MAPK members ERK1/2, JNK, and p38 were also found to be activated and depended on G*α*i in the CCR7 signaling cascades and played an important role in regulating DCs chemotaxis. Besides the role of G*α*i in the chemokine receptors signaling pathway, in recent years, chemokine receptors coupled to other G protein subfamilies has also been demonstrated. Study found that Gnaq −/− DCs were unable to migrate to inflammatory sites and LNs in vivo, which indicated the role of G*α*q in the chemokine receptor signaling. G*α*q, like CD38, regulated the extracellular calcium entry in chemokine-stimulated cells [83]. In addition to G*α*q, CCR7 also used another pathway involving Rho/Pyk2/cofilin and presumably depended on G12/G13 to control the migratory speed of DCs [81].

## 5. Perspectives

Several families of chemokines receptors and their chemokine ligands orchestrate subsets of DCs trafficking. For cDCs, CCR7 plays a central role in the migration of mature DCs to the draining LNs via lymphatic vessels during both inflammation and steady-state conditions, with a multimodule signaling model that involved G*α*i, G*α*q, and G*α*12. CXCR4, CCR2, and CXCR5 have also been implicated to be involved in some subsets of cDCs migration from periphery tissues to draining LNs. CCR2 is also implicated in control of CD8*α* ^+^ DC to the spleen and relocalization of CD11c^+^ DC from the marginal zone to the T cell areas in spleen. CCR1, CCR2, CCR5, and CCR6 are involved in the recruitment of cDCs to different tissues in specific situations. pDCs use a very different migratory patterns compared with cDCs. For pDCs, CCR5, ChemR23, CXCR3, and CCR7 are involved in migration of pDCs to LNs via hematogenous route, though the role of CCR5 versus CXCR3 and role of CCR7 in pDCs migration remains controversial. CCR7, CXCR3, and CXCR4 are shown to be involved in pDCs migration from blood to spleen. CCR9 is shown to have a role in controlling the migration of pDC to the small intestine under both steady-state and inflammatory conditions. In other situations of inflammation or tumor, CXCR3, ChemR23, and CCR6 are implicated to be involved in pDCs migration to periphery tissues.

However, there are still some limitations in the present studies on the role of chemokines receptors in the control of DCs migration. Some conclusions on the role of chemokines receptors in subsets of DCs migration came from indirect evidence by studying the expression change of chemokine receptors on DCs or by using an antagonist of a chemokine receptor to draw a possible conclusion. Experiments using chemokines receptors knockout mice also have their limitations, because it can affects a wide variety of cells potentially implicated in the inflammation. Studies using in vitro derived DCs may not accurately mirror the situations occurring in vivo. The role of chemokines receptors in the control of migration of a specific subset of DCs remains to be defined which causes the different roles of subsets of DCs in immune regulation. Understanding this complex orchestration of chemokines receptors in the subsets of DCs migration will be essential to manipulate efficiently the function of a specific subset of DCs and facilitate our clinical treatment in multiple diseases in which DCs are involved.

## Figures and Tables

**Table 1:** Chemokine receptors and chemokines involved in migration of mouse DCs subsets.

Receptor	Ligands	Cellular distribution	Role in migration	Reference
CCR1	MIP-1*γ*/CCL9	Immature DCs	Recruitment of CD11b^+^ DCs to the dome regions of Peyer's patch	[22]
			Recruitment of DC precursors into airway epithelium during bacterial inflammation and steady-state	[23]

CCR2	CCL2/MCP-1	Immature DCs	Recruitment of DCs in the lung during allergic inflammation, and supposed to be critical in inducing T(H)2 responses	[24, 25]
			Recruitment of CD11c^dim/intermediate^ DCs in the lung during mycobacterium tuberculosis infection and cDCs during Cryptococcus neoformans infection, may be important in inducing T(H)1 responses	[26, 27]
		Mature DCs	Activated LC migrate from skin to draining LNs and regulate infection-induced relocalization of CD11c^+^ DC in spleen	[28]

CCR5	MIP-1*α*/CCL3 MIP-1*β*/CCL4 Rantes/CCL5	Immature DCs mature DCs	Recruitment of pDC to inflamed peripheral lymph nodes	[29]

CCR6	CCL20/MIP-3*α*	Immature myeloid DC, subsets of pDCs	Recruitment of myeloid CD11c^+^ CD11b^+^ dendritic cells to the dome regions of Peyer's patches	[30, 31]
			Recruitment of myeloid DCs to the inflamed epithelial tissues such as skin	[32]
			mediate pDC recruitment to inflamed epithelia	[33]

CCR7	CCL19/MIP-3*β* CCL21/SLC	Mature DCs	essential for directing the antigen-loaded mature cDCs to the T cell-rich areas of the draining lymph node during inflammatory and steady-state conditions	[34]
			Migration of pDCs to LNs via HEV under both steady-state and inflammatory conditions	[35]
			Migration of pDC to the splenic white pulp under steady-state conditions	[36]

CCR9	CCL25	Myeloid and pDC	Controls the migration of pDC to the small intestine under both steady-state and inflammatory conditions	[37]

CCR10	CCL27 CCL28	Subset of tonsil pDCs, IL-3-cultured blood pDCs	Mediate pDC homing into inflamed epithelia	[33]

CXCR3	CXCL9 CXCL10/IP-10 CXCL11	pDC precursors pDC CD11c^+^ myeloid DCs monocyte-derived iDC	Migration of pDC to inflamed LNs via HEV	[38]
			migration and clustering of splenic plasmacytoid DCs in the spleen marginal zone	[39]

CXCR4	CXCL12/SDF-1*α*	Immature DCs mature DCs pDC	Migration of skin dendritic cells to LNs	[40]
			Migration of pDC to the splenic white pulp under steady-state conditions	[36]

CXCR5	CXCL13/BLC/BCA-1	Activated skin DC	Activated dermal DC migrate to draining LNs	[41]

**Table 2:** Chemokine receptors and chemokines involved in migration of human DCs subsets.

Receptor	Ligands	Cellular distribution	Role in migration	Reference
CCR1	MIP-1*γ*/CCL9	Immature DCs	May be involved in human peripheral blood monocyte-derived dendritic cells migration	[42]

CCR2	CCL2/MCP-1	Immature mature DCs	Recruitment of circulating blood DCs and monocytes to inflamed tissue	[43]

CCR3	Eotaxin eotaxin-2	Immature DCs mature DCs	May be involved in dendritic cells migration	[42, 44]

CCR5	MIP-1*α*/CCL3 MIP-1*β*/CCL4 Rantes/CCL5	Immature DCs mature DCs	Attract DCs to migrate cross the human intestinal epithelium and sample luminal virions	[45]
			May contribute to the recruitment of blood myeloid DC to cerebrospinal fluid in multiple sclerosis patients and acute optic neuritis.	[46]
			May be involved in the altered homing of blood DCs during the alloimmune response	[47]

CCR6	CCL20/MIP-3*α*	pDCs	May be involved in leukemic pDCs and blood pDCs from melanoma patients recruitment to lesions of skin	[48, 49]
			Recruitment of circulating blood DCs and monocytes to inflamed tissue	[43]

CXCR3	CXCL9 CXCL10/IP-10 CXCL11	pDCs immature CD1a^+^ DC	Might be involved in the recruitment of pDC and immature CD1a^+^ DCs to tissue lesions	[50, 51]

ChemR23	Chemerin	Immature pDCs	Migration plasmacytoid dendritic cells to lymphoid organs and inflamed skin	[52, 53]
